# Zero-Trust Access Control Mechanism Based on Blockchain and Inner-Product Encryption in the Internet of Things in a 6G Environment

**DOI:** 10.3390/s25020550

**Published:** 2025-01-18

**Authors:** Shoubai Nie, Jingjing Ren, Rui Wu, Pengchong Han, Zhaoyang Han, Wei Wan

**Affiliations:** 1 School of Computer Science and Engineering, University of New South Wales, Sydney 2052, Australia; 2 School of Computer Science, School of Cyber Science and Engineering, Engineering Research Center of Digital Forensics, Ministry of Education, Nanjing University of Information Science and Technology, Nanjing 210044, China; 3 School of Software, Shandong University, Jinan 250100, China; 4 State Grid Zaozhuang Power Supply Company, Zaozhuang 277899, China

**Keywords:** Internet of Things, zero trust, blockchain, inner-product encryption, access control

## Abstract

Within the framework of 6G networks, the rapid proliferation of Internet of Things (IoT) devices, coupled with their decentralized and heterogeneous characteristics, presents substantial security challenges. Conventional centralized systems face significant challenges in effectively managing the diverse range of IoT devices, and they are inadequate in addressing the requirements for reduced latency and the efficient processing and analysis of large-scale data. To tackle these challenges, this paper introduces a zero-trust access control framework that integrates blockchain technology with inner-product encryption. By using smart contracts for automated access control, a reputation-based trust model for decentralized identity management, and inner-product encryption for fine-grained access control, the framework ensures data security and efficiency. Firstly, smart contracts are employed to automate access control, and software-defined boundaries are defined for different application domains. Secondly, through a trust model based on a consensus algorithm of node reputation values and a registration-based inner-product encryption algorithm supporting fine-grained access control, zero-trust self-sovereign enhanced identity management in the 6G environment of the Internet of Things is achieved. Furthermore, the use of multiple auxiliary chains for storing data across different application domains not only mitigates the risks associated with data expansion but also achieves micro-segmentation, thereby enhancing the efficiency of access control. Finally, empirical evidence demonstrates that, compared with the traditional methods, this paper’s scheme improves the encryption efficiency by 14%, reduces the data access latency by 18%, and significantly improves the throughput. This mechanism ensures data security while maintaining system efficiency in environments with large-scale data interactions.

## 1. Introduction

The emergence and defining features of the zero-trust security model represent a pivotal advancement in the evolution of modern network security. This model originated from the concept of de-perimeterization proposed by the Jericho Forum in 2004, challenging the traditional security defense model centered on fixed boundaries. The zero-trust model has profoundly disrupted traditional security strategies. At its core, the principle of “never trust, always verify” [1] underpins the zero-trust model, driving a fundamental transformation in access control and identity management methodologies. The zero-trust approach to access control mandates that users and devices be restricted to accessing only the information and resources required for the completion of their specific tasks. This permission control is not limited to the initial access to the network but also includes dynamic permission adjustments during the session. The introduction of zero-trust self-sovereign identity (SSI) management further strengthens the zero-trust model. In the zero-trust framework, access control emphasizes verifying identities for every access. By providing a decentralized method of identity verification, each device and user holds an independently controlled, decentralized digital identity. This approach guarantees that resources are accessible solely to authorized entities, thereby strengthening system security, improving transparency, and enabling a more reliable authentication process. The document protects data confidentiality and integrity through data encryption, preventing data leakage or tampering. Real-time traffic monitoring enables the analysis of network activity, the detection and filtering of abnormal access, and the prompt mitigation of potential security threats. Similarly, application segmentation and control restrict application access and operational boundaries, thereby minimizing security vulnerabilities [2]. Security analysis comprehensively detects and analyzes networks, systems, and applications, identifying vulnerabilities and threats, thereby providing strong support for policy enforcement. Policy enforcement entails formulating tailored security measures grounded in the system’s specific conditions and vulnerabilities, ensuring that the overall network operates securely and stably [3]. As an advanced cybersecurity concept, zero trust provides robust protection for modern network security through its unique features and core capabilities [4,5]. It breaks the limitations of traditional perimeter security, achieving comprehensive, dynamic, and granular management of network security [6].

In real-world applications, the components of the zero-trust model, such as micro-segmentation and software-defined perimeters, are highly relevant. For instance, in smart cities, robust security measures are required to manage vast networks of interconnected devices. In this context, zero trust’s decentralized identity management and dynamic access control help ensure that only authorized entities can access sensitive infrastructure data, such as traffic systems and utilities, thereby preventing unauthorized access that could jeopardize the city’s operations. Similarly, in smart homes, where various IoT devices interact to control everything from lighting to security systems, zero trust ensures that only authenticated devices and users can access sensitive home networks. In the industrial IoT sector, where machines, sensors, and devices are critical to operations, zero trust and self-sovereign identity (SSI) can effectively authenticate and control access to industrial control systems, mitigating the risk of cyberattacks that could disrupt operations or cause physical harm.

In 6G networks, a multitude of devices, functions, processes, and communication data, whether in the physical or virtual world, need to meet the requirements of time criticality, scalability, reliability, and security [7,8]. To address these challenges, 6G networks will rely on distributed and decentralized infrastructure and management. With the emergence of a multitude of devices and digital twins, zero-trust solutions that rely on decentralized or hierarchical distributed learning may become an alternative to the current distributed solutions that heavily depend on centralized management [9]. Device authentication, communication data verification, and the ongoing monitoring of IoT data in virtual environments and the metaverse are critical steps in the process of data sharing and access [10]. Recent advancements, such as hybrid blockchain models [11], lightweight encryption algorithms [12], and adaptive trust mechanisms [13], have further enhanced decentralized access control. These approaches enable better scalability and efficiency, which are crucial for IoT applications in 6G environments. Therefore, decentralization is the key to achieving true zero trust [14]. Solutions should provide distributed key management, distributed access control management, and identity management without third-party involvement. This shift towards decentralization will require critical support such as blockchain, homomorphic encryption, and self-sovereign identity management [15].

Blockchain, a type of distributed ledger technology, offers decentralized and tamper-resistant capabilities for managing data [16]. In a 6G network environment, this technology ensures data authenticity and integrity, providing stronger support for the zero-trust security model [17]. Inner-product encryption, a specific form of functional encryption, features partial decryption capabilities, enabling fine-grained access control. Additionally, inner-product encryption can better protect users’ privacy by concealing access control attribute policies through the calculation of vector inner products [18]. Blockchain and inner-product encryption meet the zero-trust model’s demands for decentralized management and the principle of least privilege, which are particularly important in 6G networks.

In summary, as the next-generation network evolves, the interaction of data among IoT devices will face increasing risks. To overcome these challenges, this paper presents a zero-trust access control framework leveraging blockchain technology and inner-product encryption:

(1) This paper introduces a zero-trust access control mechanism built upon blockchain and registered inner-product encryption, utilizing smart contracts to define software-based boundaries across various application domains. Through the Policy Decision Point (PDP), different Policy Enforcement Points (PEPs) are allocated, allowing only verified users to access their authorized resources, thereby significantly reducing the risk of network attacks.
(2) A self-sovereign identity management system (SSIM) is proposed, utilizing node reputation values to build a consensus trust model. Combined with blockchain technology and Shamir’s secret sharing technique, it creates an innovative identity verification and management framework, specifically addressing identity security and data privacy protection in decentralized environments. The system achieves the secure uploading, storage, and verification of identity data through a dynamic consensus mechanism and distributed node storage. Through the use of reputation value consensus to construct a trust model for user identities, the approach guarantees the integrity and security of identity data without dependence on centralized certification authorities and while improving the system’s attack resilience and fault tolerance. Furthermore, the system incorporates fine-grained access control to regulate permissions for different data requesters, ensuring that sensitive information is accessible exclusively to authorized users. This not only enhances user autonomy over their identities and data but also contributes to a more efficient identity management system.
(3) Data from different application domains are stored on blockchain auxiliary chains, with the main chain storing only identity information and access logs, optimizing blockchain storage efficiency to handle high-speed data interactions in a 6G environment. Storage areas are divided based on the confidentiality levels specified by the trust mechanism, facilitating micro-segmentation, minimizing exposure to potential threats, and restricting the range of malicious activities across the system.

The structure of this paper is as follows: Section 2 provides an overview of the related work. Section 3 explores the difficulties associated with IoT access control in the context of a 6G environment. Section 4 presents the architecture and operational workflow of the zero-trust access control mechanism. Section 5 offers an in-depth explanation of the development process for the self-sovereign identity management mechanism. Section 6 presents a comprehensive comparison of experimental data. The paper concludes in Section 7, which also discusses potential avenues for future research.

## 2. Related Work

### 2.1. Traditional IoT Access Control

The substantial increase in IoT devices has imposed excessive loads on centralized network nodes. Behrad et al. [19] proposed a slice-specific mechanism for authentication and access control, leveraging virtualization technology to offload these processes for IoT devices to third-party entities. This approach alleviates the burden on the core network of connectivity providers while improving the modularity and adaptability of 5G networks. A privacy-preserving scheme for authentication, authorization, and key negotiation, based on elliptic curve cryptography, was introduced by Shin et al. [20] for wireless sensor networks in 5G-integrated IoT. This approach effectively addresses the challenges of anonymity loss and security vulnerabilities in existing sensor nodes. Wang et al. [21] proposed a tag-based lightweight access control scheme that authenticates fog nodes through the verification of shared file integrity, with embedded tag values. This approach safeguards IoT caches against unauthorized access and potential damage while ensuring the cache’s reliability and security through verifiability, efficiency, and precision. Li et al. [22] developed an access control scheme for mobile healthcare in 5G-enabled mobile industrial IoT that supports traceability and revocation. By optimizing ciphertext-policy attribute encryption, the scheme effectively protects personal health records while reducing computational costs and storage overhead.

### 2.2. IoT Access Control in the 6G Environment

Zhou et al. [23] proposed a method to address vehicle identity verification and security issues in VANETs. This approach leverages identity-based encryption techniques to implement access control in vehicular ad hoc networks (VANETs) by authenticating vehicles and detecting a range of network attacks, including Distributed Denial of Service (DDoS) attacks. Furthermore, deep learning-based methods are utilized to identify and filter malicious packets, thereby safeguarding personal data from potential leakage. To overcome the limitations of traditional blockchain-based access control approaches in heterogeneous device environments, Wei et al. [24] introduced a trustworthy access control method for 6G-MEC networks. They demonstrated how to utilize blockchain in the proposed blockchain-assisted multi-access control method, and they introduced a trusted access control method for multi-access edge computing (MEC) based on a multi-layer blockchain architecture and a reinforcement learning-based ED weight determination algorithm, aiming to mitigate the impact on blockchain consensus behavior. Sourav Saha et al. [25] introduced an innovative access control system based on smart contracts, leveraging blockchain technology to strengthen the security of medical data processing and management. Through a detailed security analysis and experimental results, the proposed solution’s effectiveness in defending against potential active and passive attacks was verified, and it demonstrated low computational and communication costs. Ma et al. [26] proposed a novel spread spectrum-based slotted ALOHA access method that leverages the use of random non-orthogonal codes to reduce collisions and enhance the system’s capacity to accommodate more users. Without relying on sensing or power control, this method offers significant advantages over existing non-orthogonal multiple access methods in terms of enhancing user load and system flexibility.

### 2.3. Zero-Trust-Based IoT Access Control

To ensure the reliable operation of power IoT devices, Wu et al. [27] introduced a zero-trust access control approach, developing a framework that addresses four key components: user authentication, device trust, application integrity, and traffic baseline. The method incorporates attribute encryption and trust mechanisms to enforce terminal security protection. Liu et al. [28] introduced a decentralized IoT zero-trust access control mechanism using smart contracts and consensus mechanisms to effectively prevent spam and reduce reliance on centralized cloud servers. To address the hacking risks in vehicular networks, Fang et al. [29] proposed an integrated security framework that combines multi-factor authentication, state-secret algorithm encryption, and blockchain technology. By employing a zero-trust network architecture, the solution enables dynamic access control and the real-time monitoring of user behavior, thereby significantly improving the stability and security of the data transmission system. Dhara et al. [30] introduced a security framework for IoT devices that combines zero-trust principles with blockchain technology. This framework counters IoT security threats effectively by employing risk-based network segmentation, extending the trust scope, and enhancing device identification and access control capabilities. Awan et al. [31] developed a solution that integrates a zero-trust architecture with an attribute-based access control mechanism, enabling the monitoring and facilitation of communication among IoT devices through a dynamic behavioral analysis and adaptable policy enforcement. Additionally, blockchain technology is employed to safeguard user attributes and ensure data security. To tackle the efficiency challenges of blockchain in IoT applications, Wang et al. [32] proposed a zero-trust-based data storage solution for IoT, which integrates sharding technology with pluggable vector commitments, replacing traditional Merkle trees. This approach reduces communication overhead and enhances the stability and security of the IoT system.

### 2.4. Decentralized Identity Management in the Internet of Things

As the Internet of Things continues to evolve, a new paradigm is emerging that facilitates autonomous interactions between machines. The future of IoT is poised for decentralization, necessitating that users serve as the root of trust for their devices, thereby enabling an owner-centric IoT ecosystem. Sandro Rodriguez Garzon et al. [33] introduced a decentralized identity management approach for 6G networks, enabling secure mutual authentication between network entities across different trust domains, independent of a trusted third party. This approach enables network entities to establish and enhance trust relationships across domains through the exchange of information. Xu et al. [34], building on the foundation of the Internet of Things, described a more general approach, achieving user authentication through distributed network-based identity management. Their approach completely separates user identity information from the data required for authentication to the mobile network. The latter type of data is stored across a distributed network, allowing users to access the mobile network while preserving their privacy and preventing the disclosure of personal information. In the Internet of Things, the distinctive characteristics of IoT nodes present not only familiar challenges but also additional complexities for identity management [35]. The benefit of distributed identity management lies in enabling users to generate, store, and authenticate their own keys, thereby verifying their identities independently. Xiong et al. [36] introduced a blockchain-based decentralized identity management solution to resolve the challenges associated with device and user identity management in large-scale IoT environments. Smart contracts are employed to facilitate large-scale user access control, while trust management techniques, including reputation assessment and credit penalty mechanisms, are developed to mitigate various attack types in distributed identity management scenarios.

### 2.5. Comparison and Contribution

Although many existing solutions integrate blockchain and encryption systems into IoT access control, the approach proposed in this paper differs significantly from previous solutions in several key aspects. Firstly, this paper introduces a novel combination of a zero-trust architecture with a decentralized self-sovereign identity management mechanism, enabling dynamic and autonomous identity verification without relying on centralized authorities. Secondly, it employs a registration-based inner-product encryption technique that eliminates the need for a key generation center, providing fine-grained access control and enhancing data security, thereby addressing the main limitations of traditional encryption schemes. Thirdly, the integration of blockchain sidechains optimizes data storage by segmenting application domains and reducing the load on the main chain, which improves the system’s scalability and throughput. Finally, the trust mechanism dynamically updates access policies based on real-time behaviors and long-term trends, ensuring stable performance, even in volatile IoT environments. These innovations set the proposed method apart from existing solutions, highlighting its advantages in addressing security and efficiency challenges in IoT within 6G networks.

## 3. Problem Discussion

In the future 6G network environment, IoT devices will continue to expand and diversify rapidly, bringing unprecedented challenges. As a hyper-scale network, 6G demands a highly scalable security architecture [37]. However, traditional security strategies may not only be costly but may also not be applicable when facing such a vast scale. Furthermore, the heterogeneity of 6G enables multiple network operators with different management architectures and signaling systems to jointly manage the network. This requires a security architecture capable of effective collaboration across different control domains, a demand that traditional centralized architectures may struggle to meet. Additionally, the primary endpoints in 6G networks are machines and devices, which exhibit significant differences in functionality and resources. Hence, a lightweight security architecture is necessary.

In this context, traditional access control and network security approaches encounter substantial challenges, making the zero-trust security model an attractive alternative for ensuring data security, owing to its enhanced scalability and flexibility [38]. Firstly, the increasing severity of security threats brought about by network openness may render traditional network security measures ineffective. Therefore, it is essential to incorporate the zero-trust security model and develop novel authentication and encryption systems to strengthen network security. Moreover, the integration of 6G into non-terrestrial environments, including satellite and maritime communications, introduces new security challenges that necessitate the development of innovative open authentication methods [18]. Furthermore, as technology progresses, users increasingly require authentication methods that offer enhanced security and greater convenience. This necessitates 6G networks to provide more intelligent and efficient authentication mechanisms, a requirement that can be met by the dynamically adaptive access policies of the zero-trust model.

While existing zero-trust architectures have enhanced network security to some degree, they fall short of addressing the complex security challenges posed by 6G networks [39]. First, although the granular access control policies of zero-trust architectures ensure the protection of data resources and computing services, they may result in considerable performance overheads in the hyper-scale networks of 6G. Second, zero-trust architectures, originally designed for networks with a centralized logical controller, struggle to accommodate the decentralized management and heterogeneity inherent in 6G networks. Furthermore, the mandatory end-to-end encryption required by zero-trust architectures makes it challenging for resource-limited 6G IoT terminals to meet their security needs. In addition, existing zero-trust architectures focus mainly on preventing data leaks and lateral movements within the network and are less effective against new types of threats such as flood attacks that 6G networks might face. Therefore, there remains a critical need to refine and optimize the existing zero-trust architecture to better support IoT access control in the context of 6G environments.

In the 6G IoT environment, current decentralized identity management solutions are particularly inadequate in meeting the demands of future network environments. While decentralized technologies offer some level of security and data privacy, they still face numerous challenges in the large-scale and highly dynamic 6G IoT environment. Firstly, traditional decentralized identity management systems often exhibit inefficiencies when dealing with a large number of devices and user identities. This is primarily due to the fact that each authentication process involves intricate interactions and consensus mechanisms, which can lead to considerable delays and resource consumption in the fast-paced, highly connected 6G environment. Moreover, as the variety and capabilities of IoT devices continue to grow rapidly, ensuring a robust and up-to-date identity verification system becomes progressively more difficult. In the absence of a centralized trust anchor, the authentication and trust establishment between devices and services often lack unified standards and protocols, leading to fragmented identity management and hindering global security and interoperability.

Therefore, zero-trust security architectures are especially critical in this environment. The identity management mechanism within a zero-trust environment must be capable of real-time response and validating diverse identity requests, ensuring that only fully authenticated users or devices are granted access to network resources.

Self-sovereign identity emphasizes that individuals have complete control over their identity information in the identity management process, without relying on any intermediaries. In the context of 6G IoT, self-sovereign identity offers users and devices a more secure and adaptable approach to managing and sharing their identity data, thereby significantly strengthening the security and privacy protection across the network. With technologies like blockchain, SSI can provide a decentralized yet efficient identity verification mechanism while maintaining user privacy, aligning with the security and operability requirements under the principle of zero trust.

Blockchain technology, a type of distributed ledger, provides robust, tamper-resistant, and decentralized data management capabilities [40]. In a 6G network environment, the application of blockchain technology ensures data authenticity and integrity, thereby reinforcing the zero-trust security model. Blockchain ensures that all data changes within the network are verified and confirmed by network participants through its consensus mechanism, reducing reliance on a single centralized management entity and enhancing the system’s tamper resistance and transparency [41,42]. Blockchain can securely manage and authenticate device identities, which is crucial for the large-scale deployment and interoperability of devices in 6G networks. In 6G, devices or services can be authenticated through the blockchain, with each device’s status updates or service requests recorded and verified on the blockchain, thus achieving true end-to-end security. Furthermore, blockchain can support smart contracts, automatically executing network policies or security protocols, thus further enhancing network automation and security.

Integrating blockchain and inner-product encryption technologies to establish a zero-trust security model in the complex and dynamic IoT environment can foster a more secure, reliable, and decentralized 6G network. This approach supports large-scale device interconnectivity and efficient data processing while simultaneously ensuring robust security and privacy protections.

## 4. Zero-Trust Access Control Mechanism Based on Blockchain and Inner-Product Encryption

### 4.1. Zero-Trust Security Model in the Context of 6G and IoT

**Figure 1:**
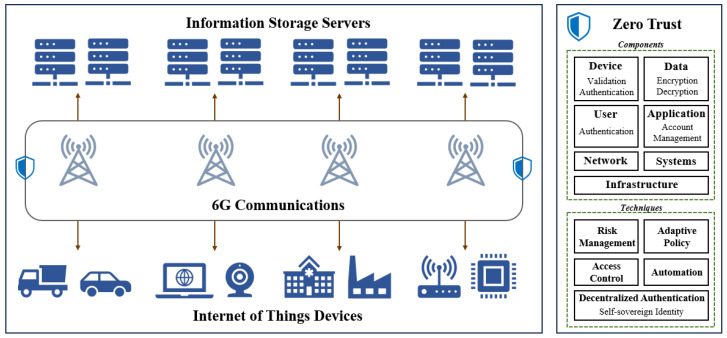
Zero -trust security model in 6G.

In the 6G environment, the exponential growth of IoT devices and the rapid expansion of network scale pose significant challenges to traditional security models, particularly in terms of access control and identity management [43]. The ultra-large-scale and highly heterogeneous network environment requires security architectures to not only have extremely high flexibility and scalability but also to adapt to decentralized management and diverse systems [44]. Additionally, the extensive differences in functionality and resources among IoT devices further exacerbate these challenges, necessitating innovation and the optimization of existing security strategies. To address these challenges, this paper introduces a zero-trust self-sovereign identity management and access control framework that leverages blockchain technology and inner-product encryption. It employs smart contracts on the blockchain to define software-based boundaries across various application domains, enabling authenticated users to access resources within their authorized scope, thereby facilitating access control. Simultaneously, it leverages a trust model built on user reputation values to achieve decentralized self-sovereign identity management, with the specific model depicted in Figure 1.

Figure 1 depicts the zero-trust security model in the 6G environment, highlighting IoT devices and information storage services. It also outlines the comprehensive process of ensuring data access security and managing user identities throughout the entire workflow, utilizing a range of technologies. In the context of permission management, the zero-trust model follows the principle of least privilege, ensuring that users and devices are granted only the essential permissions necessary to perform their tasks [45]. This design limits the scope of damage that attackers could potentially cause upon successful penetration. Simultaneously, the model strengthens internal security by partitioning the network into multiple isolated security zones, each corresponding to distinct application domains [46]. This is achieved through micro-segmentation strategies, which effectively limit the potential for lateral movement within the network. Regarding authentication and management, this paper introduces self-sovereign identity to enhance the security and autonomy of users during identity verification. Leveraging blockchain technology, self-sovereign identity guarantees the immutability of identity data and enables users to disclose only the minimal information required by requesting parties, in accordance with the principle of least privilege within the zero-trust model. Combining multi-factor authentication, this model not only enhances the reliability of identity verification but also effectively mitigates security risks through continuous monitoring and smart contracts, providing robust protection for network security. This zero-trust model, integrating SSI and multi-factor authentication, offers an efficient and dynamic security framework for identity management and data protection in the 6G network environment. Furthermore, the zero-trust model prioritizes the continuous monitoring of network activities and access to resources. Through trust mechanisms embedded in smart contracts for access behavior analysis and anomaly detection systems, any abnormal behavior can be promptly identified and addressed. Moreover, the application of smart contract automation technology reduces the need for manual intervention, thereby enhancing the speed and efficiency of security incident responses [47].

Regarding the data transmission and storage aspect of the zero-trust model, this paper consistently ensures that data remain encrypted using inner-product encryption algorithms, thereby safeguarding data security and privacy. Even in untrusted environments, data are effectively protected, preventing data leakage and misuse. Identity management and access control are essential components of the zero-trust model [48]. By employing a trust model based on user reputation values for dynamic consensus and utilizing distributed node storage, the secure uploading, storage, and verification processes of identity data are achieved. Access permissions are then controlled according to detailed policies. This fine-grained management approach effectively mitigates unauthorized access and data leakage risks. Privacy design is an integral part of the zero-trust architecture. From the outset, it fully considers the privacy protection needs of individuals and organizations, ensuring that data are always respected and protected throughout the collection, processing, and usage processes.

Implementing zero trust requires a series of rigorous steps. First, it is necessary to identify and categorize key data and resources, understanding how data flow within the network. Next, precise access control policies are established, and corresponding security technologies are deployed. Finally, through continuous monitoring and analyses, suspicious behaviors are detected, and a swift response is enacted upon detecting security incidents. In the implementation process, continuously adjusting and improving security policies and measures is also crucial to address the ever-changing security threats and business needs.

### 4.2. The Zero-Trust Security Framework

**Figure 2:**
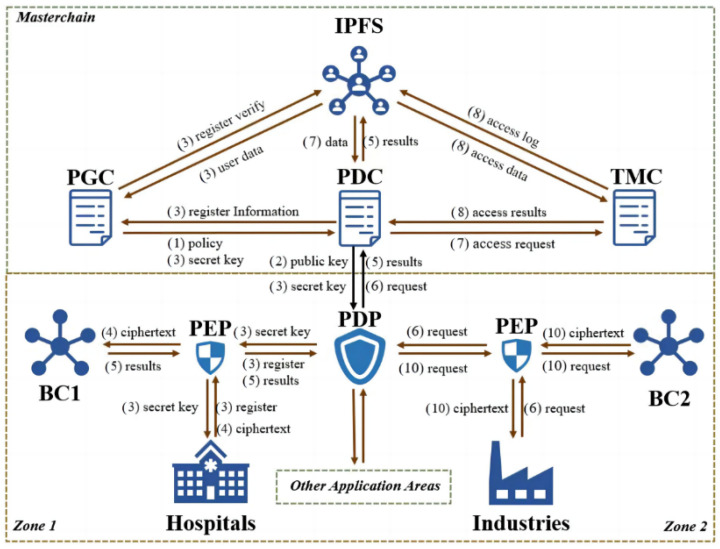
The zero-trust security framework.

The zero-trust security framework presented in this paper is depicted in Figure 2. The upper layer’s main chain consists of four components that collectively form the PDP (Policy Decision Point) in the zero-trust framework. These components include the InterPlanetary File System (IPFS), the Policy Generation Contract (PGC), the Policy Decision Contract (PDC), and the Trust Mechanism Contract (TMC). The PDP (Policy Decision Point) is responsible for dynamically adjusting access control policies for different application domains, conducting a real-time analysis of the requesters’ identities and permissions and the requested resource types to ensure the security of heterogeneous device data. The access control domain in the lower layer is primarily composed of three components: the Policy Enforcement Point (PEP), the sidechain (BC), and the data entities within the application domains. The PEP dynamically enforces access policies issued by the PDP in real time, providing access services to different data subjects through the sidechain. This design allows the proposed framework to fully leverage the decentralization and tamper-resistant features of blockchain while ensuring fine-grained access control through inner-product encryption. The following describes the specific functions of these components and their roles in practical implementation.

**IPFS:** On the main chain, the IPFS functions as a distributed storage system, housing the identity information, trust ratings, and access logs of users from different application domains, as well as the associated access control policies and trust lists. This ensures the scalability of the framework by separating identity and access information from large-scale data storage, enabling faster access to trust records during system operations.

**PGC:** Positioned on the main chain, the PGC is a smart contract responsible for generating specific access control policies for different domains. It reads user trust values and levels in real time and dynamically adjusts policies, formulating fine-grained strategies based on domain characteristics and data confidentiality levels. It also manages the generation of key pairs for inner-product encryption, ensuring that encryption and decryption keys are securely and dynamically distributed across devices without requiring a centralized key management authority.

**PDC:** Also on the main chain, the PDC is a smart contract that processes access requests and security policies relayed by the PDP in real-time, acting as the actual control program for the PDP. The PDC evaluates access requests against predefined policies and uses the trust values computed by the TMC to make dynamic access decisions, ensuring real-time adaptation to changing security requirements.

**TMC:** Hosted on the main chain, the TMC manages and calculates the trust values and levels of user entities. This contract considers various behavioral histories of devices recorded in the ledger, extracting multiple key factors to compute the trust levels of different devices. This process enables the system to dynamically modify trust levels according to the actual behavior of devices. This improves the system’s overall security and trustworthiness. In addition, the TMC integrates with the inner-product encryption scheme to validate access permissions by comparing encrypted policy vectors with attribute vectors, further reinforcing the framework’s zero-trust principles.

**PDP:** An essential component of the zero-trust architecture, the Policy Decision Point (PDP) evaluates access requests based on predefined security policies, determining whether users can access specific resources. It assesses user identity, contextual factors, and security policies to make access control decisions, ensuring that only authorized individuals can access sensitive resources.

**PEP:** Within the zero-trust framework, the PEP implements access control policies. Situated between users and resources, it oversees access requests and grants or restricts access according to the authorization decisions made by the PDP. By implementing fine-grained access control policies, the PEP effectively protects system resources from unauthorized access and malicious activities. It also serves as the interface between IoT devices and the blockchain, facilitating seamless communication and ensuring the enforcement of dynamically updated access policies.

**BC:** The BC is an auxiliary storage chain in different application domains, primarily used for storing data on subjects’ information. With the assistance of multiple auxiliary chains, it effectively enhances the access control system’s efficiency while reducing the main chain’s load and providing storage isolation. By isolating data storage based on application domains, the BC ensures that sensitive data are encrypted and categorized using inner-product encryption before being stored, providing an additional layer of security.

**Application Domain Entities:** Users are segmented based on their application domains, such as healthcare, industry, and home. These domains rarely need to share data; dividing them enhances data availability and significantly reduces system maintenance pressure. Each domain establishes distinct security access policies based on the confidentiality of the stored data and the trust levels associated with user roles. This approach further accommodates the diversity and heterogeneity of IoT devices, resulting in a more granular and effective access control mechanism.

By combining blockchain’s decentralized ledger with inner-product encryption’s fine-grained access control, the proposed framework ensures both data security and operational efficiency in real-world IoT environments. Blockchain enables transparent, tamper-resistant storage of access logs and trust ratings, while inner-product encryption dynamically evaluates access permissions based on attribute policies, ensuring compliance with zero-trust principles. This combination provides robust support for the framework’s implementation in diverse application domains.

**Preparation Phase:** (1) The Policy Generation Contract (PGC) formulates appropriate security policies based on the domain-specific entities and the confidentiality of the data, and it sends these policies to the Policy Decision Contract (PDC). (2) Upon receiving the security policies, the PDC stores them in the InterPlanetary File System (IPFS) and, referencing the user trust list maintained by the Trust Mechanism Contract (TMC), generates initial inner-product encryption key pairs through these policies, which are then distributed to the Policy Enforcement Points (PEPs) in various domains via the Policy Decision Point (PDP). (3) Each PEP develops a plan based on the dispatched access control policies. Every new user or IoT device must undergo key registration and attribute upload, during which the PEP returns the corresponding auxiliary keys based on the attributes and new public key of each newly registered entity. (4) When application domain entities need to store data, they authenticate through the PEP, which, after validating the storage entity’s identity through the PDP, categorizes the uploaded data according to the access strategy, rates the data for confidentiality, applies encryption measures, and uploads the encrypted data to the auxiliary chain (BC). To manage the scalability of millions of IoT devices in a 6G environment, the auxiliary chain uses a domain-based segmentation approach, where each domain corresponds to an independent chain. This allows data and requests to be processed locally within the corresponding auxiliary chain, significantly reducing the load on the main chain and improving system efficiency. (5) The framework also incorporates a dynamic load-balancing mechanism to distribute requests across multiple auxiliary chains based on current system loads and resource availability. This ensures that no single chain becomes a performance bottleneck. As the number of IoT devices grows, new auxiliary chains can be added horizontally, allowing the system to scale seamlessly while maintaining high performance and low latency in data processing. After successful data upload, the PEP returns the storage results and information to the PDP, which then stores the corresponding information on the main chain.

**Execution Phase:** (6) When users or IoT devices need to access data, they first send data requests to the PEP, which then uploads the requests to the PDP. (7) Upon receiving the requests, the PDP first checks the legality of the access by comparing the unique identifiers of the access entities in the IPFS database. If the access is legal, the PDP forwards the request to the TMC. (8) The TMC calculates the trust value of the accessing entity based on existing access records and trust levels in the IPFS. After completing the calculations, it stores the current access record in the IPFS, updates the trust value in real time to the trust table, and returns the computed results to the PDC. (9) The PDC, after a comprehensive assessment of all information, returns the processed results to the PEP via the PDP. (10) The PEP, based on the results, sends data requests to the auxiliary chain, retrieves the corresponding encrypted data segments, and returns them to the requesting entities. Users or IoT devices then decrypt the data offline using the keys issued at registration.

In this process, blockchain technology facilitates a secure, transparent, and anonymous communication environment for diverse Internet of Things (IoT) devices. To ensure the security of these devices and their data, this mechanism employs inner-product encryption for data encryption and utilizes smart contracts to finely manage the communication processes between devices. When the Policy Decision Point (PDP) receives a request to establish new policies, it triggers the Policy Generation Contract (PGC) to run, formulating a new set of access strategies for the inner-product encryption algorithm.

To systematically record and manage the attributes of IoT devices and the data that they generate, this mechanism employs InterPlanetary File System (IPFS) technology. The outstanding features of the IPFS enable it to easily handle the challenges of storing large files, effectively addressing the limitations imposed by blockchain size. Furthermore, the IPFS provides a secure and reliable storage solution through automated resource allocation and data fingerprinting. Notably, the IPFS is tightly integrated with smart contracts, allowing the authenticity of the information stored on the IPFS to be validated by cross-referencing it with transaction data on the blockchain ledger, thereby ensuring the accuracy and integrity of the data.

During the data-sharing process, the PDP smart contract approves or denies communication requests from IoT devices based on the calculated trust levels. If a device’s trust level meets the requirements, its communication request will be approved; otherwise, it will be denied. Such a design allows the system to ensure security while facilitating efficient communication between devices, providing robust support for the development of the IoT.

To further highlight the functional advantages of the proposed zero-trust access control mechanism over existing schemes, we provide a detailed comparison in Table 1. This table evaluates key aspects, including fine-grained access control, cross-domain support, data storage and transmission efficiency, and the flexibility of dynamic identity management.

The table clearly demonstrates that, compared to traditional centralized schemes and existing blockchain-based zero-trust schemes, the approach proposed in this paper exhibits significant advantages in fine-grained control for dynamic identity management, cross-domain capability, data transmission efficiency, and flexibility. The auxiliary chain-based zero-trust mechanism proposed in this study not only alleviates the burden on the main chain but also substantially enhances management efficiency in dynamic environments.

### 4.3. Three Types of Smart Contracts in the Policy Decision Point

**Figure 3:**
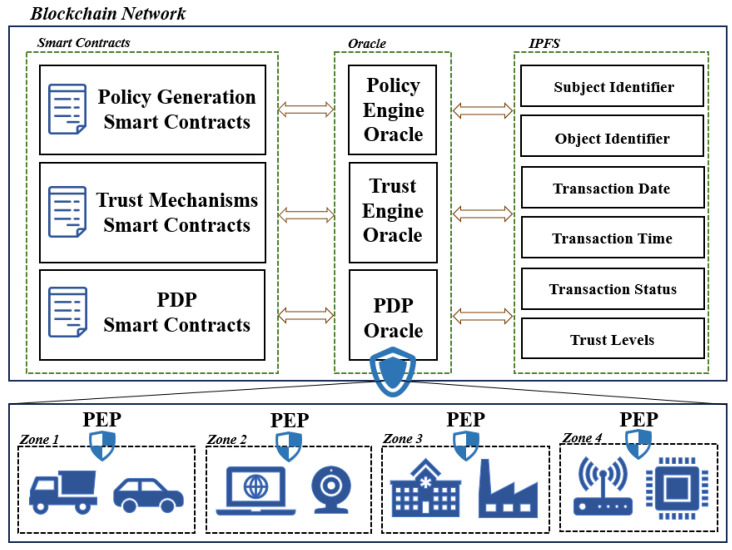
Detailed interactions of smart contracts.

As shown in Figure 3, the main chain contains three primary types of smart contracts, namely, the PGC, PDC, and TMC. Each of these contracts has its own database. Meanwhile, within the blockchain, the distributed storage system shares relevant information about data subjects, such as identity identifiers, transaction records, and trust values. When it is necessary to evaluate access requests or update trust values, the contracts retrieve the corresponding data from the distributed storage system and store the results in their respective databases. Through real-time interaction among the three contracts, the PDP can allocate different security policies to multiple PEPs in various application domains. The following section offers a comprehensive overview of the functions and their interrelationships.

PDC Contract: This contract interacts directly with the PDP and can verify the legality of data access requests by querying target identity information stored in the IPFS. It also decides whether access should be granted through interaction with the TMC. In this process, the registration of users and IoT devices is primarily conducted through this contract. The main functions included are as follows:

Init Storage: This function handles the recording of the access logs and identity details of target entities on the blockchain while also creating relevant data indices. Init Registered: This function verifies the user’s public key and unique identifier uploaded during registration for availability and legality. If valid, it sends compliant and available user attributes and public keys to the PGC to generate corresponding security policies and keys, and it uploads the user’s unique identifier to the IPFS. Access Judge: This function retrieves user information to verify identity legality; then, it interacts with the TMC to invoke the Trust Calculation function within the TMC contract to determine whether the trust level meets the data access policy requirements, and it determines whether nodes can normally store or access data resources.

PGC Contract: This contract is primarily responsible for formulating corresponding security policies for PEPs in different domains and generating inner-product encryption key pairs for users. The PGC contract maintains a list for registered-type inner-product encryption key generation and an access policy list. The functions included are as follows:

Encryption Algorithm: This function is mainly responsible for generating corresponding key pairs and auxiliary keys for users, and it needs to be invoked during user registration. Policy Generation: This function generates corresponding security policies for different application environments and formulates access policies based on user attributes. The formulated security and access policies are returned to the PEPs and corresponding users through the PDC.

TMC Contract: This contract hosts the trust mechanism, primarily calculating trust values based on users’ initial attributes and subsequent access records. The functions included are as follows:

Trust Calculation: This function is responsible for reading user information and access records from the IPFS to calculate trust values. After the calculation, it returns the trust value to the Trust Decision function and uploads the latest trust value to the IPFS for storage. Trust Log: This function receives access requests from the PDC contract, calls the Trust Calculation function to determine trust values upon receiving a request, and further returns the results to the Access Judge function. This function also enables the retrieval of the most recent trust values and honesty assessments of nodes. Trust Division: This function allocates storage areas of corresponding confidentiality levels to data when users or IoT devices store data, based on user attributes.

### 4.4. The Zero-Trust Security Framework

#### 4.4.1. Definition of Identity-Based Inner-Product Encryption

Identity-based inner-product encryption (IBIPE) is a cryptographic scheme that allows users to generate their own keys and subsequently register the associated public key and attributes within a smart contract. The smart contract aggregates individual user public keys into a unified, compact master public key. In some cases, users may need to retrieve a secondary decryption key from the smart contract, which is then combined with their own private key for decryption. The entire key management process is designed to be fully transparent, ensuring that no secrets are stored.

The definition of IBIPE is grounded in the registered inner-product encryption (RIPE) scheme introduced by Francati et al. [49]. Let n=n(λ) be a polynomial function in λ, and let *q* represent a prime number. A RIPE scheme with message space *M* and attribute space *U* is defined by the following polynomial-time algorithms:

- **Setup**$\left(1^{\lambda },1^{n},1^{L}\right)$: Given the security parameter $1^{\lambda }$, the vector length *n*, and the number of slots *L*, the setup procedure randomly generates a common reference string crs, which serves as a foundation for further cryptographic operations.
- **KGen**(crs,i): Upon receiving the public reference string crs and the slot index i∈[L], the key generation algorithm produces a public key ${\mathrm{pk}}_{i}$ and the corresponding private key ${\mathrm{sk}}_{i}$.
- **IsValid**$\left(crs,i,{\mathrm{pk}}_{i}\right)$: Given the reference string crs, the slot index i∈[L], and the public key ${\mathrm{pk}}_{i}$, the verification algorithm checks the validity of the key, returning a binary result b∈{0,1}. If b=1, the key is valid; otherwise, it is not.
- **Aggr**$\left(crs,{\left({\mathrm{pk}}_{i},x_{i}\right)}_{i\in [L]}\right)$: The aggregation algorithm takes the common reference string crs and a set of *L* pairs of public keys ${\mathrm{pk}}_{i}$ and associated non-zero vectors $x_{i}\in U$. It deterministically computes and outputs the master public key mpk and a set of auxiliary decryption keys ${\mathrm{hsk}}_{1},\ldots ,{\mathrm{hsk}}_{L}$.
- **Enc**(mpk,y,m): The encryption algorithm takes as input the master public key mpk, a non-zero attribute vector y∈U, and a message m∈M. It produces the ciphertext *c* as the output.
- **Dec**(sk,hsk,c): Given a private key sk, an auxiliary decryption key hsk, and a ciphertext *c*, the decryption algorithm deterministically outputs the message m∈M∪{⊥}, where ⊥ indicates a decryption failure.

#### 4.4.2. Data Access Process

The zero-trust framework presented in this paper begins by initializing the registered inner-product encryption (RIPE) scheme during the system’s setup phase. Initially, the Policy Generation Center (PGC) selects the security parameter $1^{\lambda }$, the vector length *n*, and the number of slots *L*, and then it executes the setup algorithm, $Setup(1^{\lambda },1^{n},1^{L})$, to generate the universal reference string (CRS), denoted as crs. With the CRS in hand, the PGC proceeds to the key generation phase by executing the KGen(crs,i) algorithm. This algorithm takes as input the reference string crs and a slot index i∈[L], and it outputs a public key ${\mathrm{pk}}_{i}$ and the corresponding private key ${\mathrm{sk}}_{i}$ for each of the initial users and IoT devices.

Parallel to this, the blockchain administrator plays a key role in deploying the smart contracts that facilitate the system’s operations. The administrator initiates a transaction on the blockchain to deploy the smart contract, sets the starting address as an initial environment variable, and configures the system’s parameters, ensuring that all the required parameters and configurations are correctly set up for the smooth operation of the zero-trust framework.

When everything is ready, new IoT devices and users need to register with the PGC via the PDP for normal data access. The PDC determines whether the unique identifier uploaded with the registration is unique, and, if it is legitimate and unique, then the unique identifier is stored via upload to the IPFS. In addition, the user needs to upload the attributes to the PGC; if the attributes are legitimate, the PGC will generate the corresponding attribute vectors for them based on the Attribute Vector Comparison Table. After that, the PGC will assign slots for them and determine whether the slots are available by inputting the universal reference string crs, the slot index i∈[L], and the public key ${\mathrm{pk}}_{i}$ via the $IsValid\left(crs,i,{\mathrm{pk}}_{i}\right)$ algorithm. If the slot determination bit $b\in \left\{0,1\right\}$ returns 0, it will perform the registration for the user and generate a new public key ${\mathrm{pk}}_{i}$ and a private key ${\mathrm{sk}}_{i}$, and, after completing the registration work, the PDP returns the generated key pair to the new user. As the user base expands, the initially established keys are updated to accommodate the growing system. To handle this, the PGC invokes the aggregation algorithm $Aggr(crs,{\left\{\left({\mathrm{pk}}_{i},x_{i}\right)\right\}}_{i\in [L]})$, which takes as input the universal reference string crs and the set of *L* public key and attribute vector pairs ${\left\{\left({\mathrm{pk}}_{i},x_{i}\right)\right\}}_{i\in [L]}$, where each pair consists of a public key ${\mathrm{pk}}_{i}$ and its corresponding non-zero attribute vector $x_{i}\in U$. This process produces a new master public key mpk along with *L* auxiliary decryption keys ${\mathrm{hsk}}_{1},\cdots ,{\mathrm{hsk}}_{L}$, which are used for subsequent decryption operations.

In the system’s data storage process, data generally come from environmental data monitored and collected by IoT devices or user data uploaded by users on their own initiative. After the PEP collects a certain amount of data, it will package the data to form the information m∈M and send a data storage application to the PDP, which will pass the judgement of this storage application through the TMC and then assign a storage area of the corresponding confidentiality level based on the attributes through the PGC. After that, the PDP will return the information summary to the PEP. After the storage request passes and the storage area is allocated for this information, the PEP will use the algorithm $Enc\left(mpk,y,m\right)$ to generate the ciphertext c and transmit the ciphertext for storage. After successful storage, the PEP will again feedback the storage situation to the PDP, make an uplink record of this data storage situation, and create the corresponding data index.

If a user or IoT device wants to access data, it first needs to send a data access request to the PEP. The PEP will upload this application to the PDP, and the PDP will judge whether there has been a new user registration to change the decryption key through the PGC and the TMC after judging the user’s attributes and trust level. If the user group has been expanded, the PGC will return the auxiliary key ${\mathrm{hsk}}_{L}$ based on the user’s information slot, and the PDP will store this access record in the IPFS. According to the success or failure of the access result, the TMC will adjust the user’s trust value in real time. Thereafter, the PDP returns the data index with the auxiliary key to the PEP; then, the PEP receives the auxiliary key and reads the ciphertext *c* according to the data index, and it returns the ciphertext to the user along with the auxiliary key. The user runs the algorithm Dec(sk,hsk,c) and inputs the key ${\mathrm{sk}}_{i}$ on the local end, the auxiliary decryption key ${\mathrm{hsk}}_{i}$, and the ciphertext *c*. The deterministic decryption algorithm outputs a message m∈M ∪ ⊥; at this time, the non-zero vector $x_{i}$ contained in the ciphertext can only decrypt the stored data M if it intersects with the user’s attribute non-zero vector y, and unsatisfied ciphertexts are still unavailable for decryption.

#### 4.4.3. Proof of Security for Registered Inner-Product Encryption

**Theorem** **1.** *For all λ∈N, n∈N, and L∈N, the RIPE scheme $\Pi_{\mathrm{sRIPE}}=($Setup, KGen, IsValid, Aggr, Enc, Dec) with message space M and attribute space U is complete.*

(1) $$\begin{array}{cc} P[\mathrm{IsValid}(crs,i,{\mathrm{pk}}_{i}) & =1|crs\leftarrow \mathrm{Setup}(1^{\lambda },1^{n},1^{L}),\\ \left({\mathrm{pk}}_{i},{\mathrm{sk}}_{i}\right) & \leftarrow \mathrm{KGen}(crs,i)]=1 \end{array}$$

**Theorem** **2.** *For all λ∈N, n∈N, L∈N, and i∈[L], the RIPE scheme $\Pi_{\mathrm{sRIPE}}=\left(\mathrm{Setup},\;\mathrm{KGen},\;\mathrm{IsValid},\;\mathrm{Aggr},\;\mathrm{Enc},\;\mathrm{Dec}\right)$ with message space M and attribute space U satisfies correctness. For all crs output by $\mathrm{Setup}(1^{\lambda },1^{n},1^{L})$ and all $\left\{\left({\mathrm{pk}}_{i},{\mathrm{sk}}_{i}\right)\right\}$ produced by KGen(crs,i), with the public key set ${\left\{{\mathrm{pk}}_{j}\right\}}_{j\in [L]\setminus \{i\}}$ such that $\mathrm{IsValid}(crs,j,{\mathrm{pk}}_{j})=1$ for each j, and for all m∈M, $x_{1},\ldots ,x_{L}\in U$, and y∈U such that $\langle x_{i},y\rangle =0$ for some i∈[L], the following equation holds:*

(2) $$P\left[\mathrm{Dec}\left({\mathrm{sk}}_{i},{\mathrm{hsk}}_{i},c\right)=m|\begin{array}{c} \left(\mathrm{mpk},{\left({\mathrm{hsk}}_{j}\right)}_{j\in [L]}\right)=\mathrm{Aggr}\left(\mathrm{crs},{\left(\left({\mathrm{pk}}_{j},x_{j}\right)\right)}_{j\in [L]}\right),\\ c\leftarrow \mathrm{Enc}(\mathrm{mpk},y,m) \end{array}\right]=1.$$

**Theorem** **3.** *If the scheme $\Pi_{\mathrm{sRIPE}}=\left(\mathrm{Setup},\;\mathrm{KGen},\;\mathrm{IsValid},\;\mathrm{Aggr},\;\mathrm{Enc},\;\mathrm{Dec}\right)$ is a valid RIPE scheme with message space M and attribute space U, then for any adversary A, we can define the following security game $Game_{\Pi_{\mathrm{sRIPE}},A}^{\mathrm{sRIPE}}\left(\lambda ,b\right)$, where b∈{0,1} represents a bit in the challenge.*

**Initial Phase:** Upon receiving the attribute length *n* and the number of slots *L* from the adversary *A*, the challenger samples the common reference string $crs\leftarrow \mathrm{Setup}(1^{\lambda },1^{n},1^{L})$ and provides it to *A*. The challenger also initializes a counter ctr=0, a dictionary *D*, and a set of slot indices $C_{L}=Ø$ to track potential corrupted slots.

**Pre-Challenge Query Phase:** The adversary *A* can make the following queries:

- **Key Generation Query:** The adversary specifies a slot index i∈[L]. In response, the challenger increments ctr=ctr+1, samples $\left({\mathrm{pk}}_{ctr},{\mathrm{sk}}_{ctr}\right)\leftarrow \mathrm{KGen}\left(crs,i\right)$, updates the dictionary to $D\left[\mathrm{ctr}\right]=\left(i,{\mathrm{pk}}_{ctr},{\mathrm{sk}}_{ctr}\right)$, and provides the tuple $\left(\mathrm{ctr},{\mathrm{pk}}_{ctr}\right)$ to *A*.
- **Breaking Query:** The adversary specifies an index c∈[ctr]. In response, the challenger retrieves the tuple $D\left[c\right]=\left(i^{\prime },{\mathrm{pk}}^{\prime },{\mathrm{sk}}^{\prime }\right)$ and …

## 5. Zero-Trust Self-Sovereign Identity Management Mechanism in 6G Network Environment

### 5.1. Initialize Parameters

First, determine the security parameter *l*, and then generate two large prime numbers *p* and *q*. Next, calculate N=p×q; the resulting *N* is part of the shared public and private key and serves as the modulus for encryption and decryption operations. Then, compute the Euler function ϕ(N)=(p−1)×(q−1), preparing for the generation of key exponents. The public key exponent *e* is chosen to be a positive integer less than ϕ(N), typically 65,537, ensuring that *e* is less than ϕ(N) and coprime with ϕ(N). Subsequently, calculate the private key exponent *d*, which satisfies d×e≡1modϕ(N), where *d* is the modular inverse of *e* with respect to ϕ(N). This completes the generation of the public key PK(N,e) and private key SK(N,d), which, together, constitute the key pair.

### 5.2. Identity Upload

When a user needs to authenticate, they submit an authentication request to the self-sovereign identity management system (SSIM) using their user ID. The user ID is a unique identifier that is usually pre-assigned by the system when a user first makes an identity declaration. After the application is submitted, the system first conducts a preliminary check of the user ID to verify the completeness of the information and the correctness of the format.

Each new node in the network is assigned an initial credibility score, which serves as the starting point for measuring its trustworthiness within the network. Over time, various behaviors of the node, such as successful transaction validation, transaction submission acceptance by the network, or instances of dishonest behavior, will affect the increase or decrease in its credibility score.

When a user requests authentication, the network of nodes within the blockchain makes a consensus decision to determine whether the user is trustworthy. To ensure the effectiveness and security of network decisions, the system sets a credibility threshold $R_{threshold}$, and only nodes that meet or exceed this threshold are permitted to participate in critical network decision-making processes. Upon receiving a user’s credibility score *R*, a node compares it with $R_{threshold}$. If the user’s credibility score is greater than or equal to $R_{threshold}$, the node casts a vote in favor. If it is below the threshold, it casts a vote against. This distribution of decision-making authority ensures that the network is managed collectively by nodes that have been validated and exhibit good behavior, thereby enhancing the overall security and reliability of the network. Additionally, a node’s voting power may be proportional to its credibility score, ensuring that nodes with higher credibility have more influence in network decisions.

After voting is completed, the system aggregates all the nodes’ voting results. Consensus is reached based on the weighted average of the voting results, with the specific calculation shown in Equation (3).

(3) $$V_{\mathrm{result}\;}=\frac{\sum_{i=1}^{n}w_{i}\cdot v_{i}}{\sum_{i=1}^{n}w_{i}}$$

Here, *n* is the total number of voting nodes, $w_{i}$ is the weight of the *i*-th node, and $v_{i}$ is the voting result of the node (1 for in favor and 0 for against). The system sets a clear consensus ratio *p*, typically greater than 50%. Only when $V_{result}\geq p$ is the user deemed trustworthy, and their identity verification request is accepted.

When the system returns the consensus result and the user is authenticated, the user uploads their complete identity information to the system. Before uploading their identity information, the user organizes it into a standardized data format. Then, the user encrypts these data using the public key pk provided by the system and generates encrypted information. This process is represented in Equation (4).

(4) C=k×G+Pm

Here, *G* is the base point of the elliptic curve, Pm is the point on the curve to which the plaintext message is mapped, and k×G represents the result of the base point *G* multiplied by the scalar *k*. Adding Pm to this result yields the ciphertext *C*.

The encrypted data are then sent to the system through a secure channel. Upon receiving the encrypted data, the system uses the private key sk for decryption. The decryption process involves using the private key sk to compute with the received encrypted data point *C* to recover the original information point Pm.

After decrypting the received information using the private key, the system will verify the integrity and validity of the data. This verification process includes checking whether the data have been tampered with during transmission and verifying the format and logical consistency of the data. Upon successful verification, the identity information will be stored in the system’s database, and a digital identity certificate will be generated, including a digital identity (DID) and verifiable credentials (VCs).

In the self-sovereign identity management system, the generation and verification of identity are primarily based on the DID and VC. The user first generates a public–private key pair. The system uses the user identifier to generate a unique DID identifier and creates a DID document containing this identifier, the public key, and authentication methods. The user signs this document with their private key and uploads it to the blockchain, ensuring its immutability and public availability. Simultaneously, the system issues a VC, including metadata, claims, and proof of digital signatures. The metadata describe the type and structure of the credential, the claims section details the user’s personal information, and the proof section is the issuer’s digital signature used to verify the authenticity of the credential.

### 5.3. Identity Storage

Traditional identity management systems are typically centralized and rely on one or a few authorized entities to store and manage personal identity information. If a centralized database is hacked, the information of all users can be compromised, leading to significant privacy and security issues. Moreover, identity information is often difficult to share and verify across different organizations, reducing system efficiency and increasing the cost of repeated verifications. This solution combines the system-generated DID and VC into a more comprehensive identity proof called DIDC. It then uses Shamir’s secret sharing technique to split the identity information into multiple parts and encrypts these parts. The encrypted parts are distributed and stored across different blockchain nodes. When there is a need to verify the user’s identity or access the complete DIDC data, authorized entities must collect a sufficient number of parts and use the appropriate decryption keys and secret sharing algorithms to reconstruct the original data. This approach not only achieves decentralized identity management, ensuring the security of user identity information, but also simplifies the verification process and reduces identity management costs.

In Shamir’s secret sharing technique, selecting the appropriate *n* (total number of shares) and *k* (minimum number of shares needed to reconstruct the secret) is the primary step in designing a secure and efficient system. The polynomial generation step is the core of the entire process, responsible for transforming the secret into multiple shares that will subsequently be distributed and stored across different nodes. The generated polynomial f(x) is a k−1 degree polynomial, where each coefficient, except for the constant term, is a secret *S* (the value of DID + VC), and the remaining coefficients $a_{1},a_{2,\;\;}a_{3},{\ldots a}_{k-1}$ are randomly chosen. This polynomial formula can be expressed as Equation (5).

(5) $$f\left(x\right)=S+a_{1}x+a_{2}x_{2}+\cdots +a_{k-1}x_{k-1}$$

These coefficients are selected within a finite field GF(p), where *p* is a large prime number. Random coefficients are chosen using a secure random number generator to ensure the randomness of the polynomial, which is critical to the security of the entire secret sharing scheme. The randomness of the coefficients not only prevents unauthorized parties from predicting other parts of the polynomial but also ensures that, without a sufficient number of shares, no one can reconstruct the complete polynomial and thus obtain the secret *S*.

After selecting the appropriate coefficients, for each specified value *i* (ranging from 1 to *n*, where *n* is the total number of shares), the polynomial is evaluated at *i*, resulting in si=f(i)modp, where si represents the share allocated to the *i*-th node. The use of modular arithmetic with *p* ensures that the value of each share is effectively controlled within a secure range, avoiding excessively large values and ensuring the correctness and security of the calculations.

A key step after generating the shares is to securely distribute these shares to different nodes or storage locations. The entire distribution process must ensure the security of each share, thereby ensuring the overall security and reliability of the system.

### 5.4. Identity Verification

When a verifier needs to confirm a user’s identity, they initiate a verification request. This request typically includes the user’s basic identification information, such as user ID or other relevant identifiers, as well as specific credential details that may need to be verified. The request is sent to the system responsible for identity verification, usually through secure communication channels to ensure the security and integrity of the request data.

Upon receiving the verification request, the system begins processing the shares stored on different nodes to reconstruct the user’s DID and the related VC. The first step is to collect shares from multiple nodes distributed across different geographical locations. The collected shares must undergo a strict verification process to confirm their integrity and authenticity, which typically includes checking the hash values of the shares or using other cryptographic verification methods to ensure that they have not been tampered with during storage or transmission. Once it is confirmed that the shares are complete and authentic, the next step is to use Lagrange interpolation to reconstruct the entire polynomial f(x). After successfully reconstructing the polynomial, the original secret *S* can be obtained by computing f(0).

Once the DIDC information is successfully reconstructed, the system processes this information to verify the user’s identity and the attributes or permissions that they claim. This may involve checking the validity of digital signatures, comparing the user-provided data with the data in the VC, and performing any other necessary security checks.

After the verification is complete, the system generates a verification result, typically including whether the verification was successful, the user’s identity details, and any related additional information. This result is then sent back to the entity that requested the verification, providing a clear response about the user’s identity and credential status. This process ensures transparency and traceability of the verification, allowing the verifier to take appropriate actions or make decisions based on the returned information.

### 5.5. Security Analysis of Self-Sovereign Identity Management Mechanisms

#### 5.5.1. Reputation-Based Trust Model

In a reputation-based trust model, the security of identity verification is built on a continuous evaluation of node behavior. Each node’s reputation score is dynamically adjusted based on its historical behavior. The calculation and updating of the reputation score are automated and conducted through consensus among multiple nodes, significantly reducing the likelihood of single-point tampering. Each node’s behavior is monitored by the entire network and is directly related to its reputation score. Thus, nodes are incentivized to maintain good behavior patterns to preserve or increase their reputation. Even if a node is initially attacked or manipulated, its abnormal behavior will result in a decreased reputation score, thereby affecting its influence within the network, ensuring a secure and reliable trust model. Additionally, by requiring nodes to reach a certain reputation threshold to participate in critical decisions, the system ensures that only the nodes verified as reliable can influence important network operations, preventing new or low-reputation nodes from posing significant threats to the system.

#### 5.5.2. Secret Sharing-Based Identity Management

In the identity management phase, sensitive information is split into multiple parts using secret sharing technology and distributed across different nodes. Even if some nodes are attacked or data are leaked, attackers cannot reconstruct the complete information from these fragments alone, as each single node only stores part of the information. Only by obtaining a sufficient number of shares can the original data be reconstructed using the secret sharing algorithm, ensuring the integrity and confidentiality of user identity information during storage. During identity verification, hash verification and Lagrange interpolation are used to reconstruct the information, preventing man-in-the-middle attacks or data tampering and ensuring the integrity and authenticity of the collected shares. Moreover, the process of verifying user information requires effectively executing digital signature verification to ensure that the data have not been tampered with and that their source is reliable.

#### 5.5.3. Defense Against DDoS and Replay Attacks

In the proposed architecture, DDoS attacks attempt to incapacitate the core components of the system by exhausting network resources and computational power. To counter DDoS attacks, this paper introduces a reputation-based dynamic trust model, which permits only nodes with reputation values exceeding a predefined threshold to participate in critical network decisions and access resources. This mechanism restricts low-reputation and malicious nodes from consuming system resources, thereby mitigating the impact of such attacks. Furthermore, by leveraging the distributed nature of blockchain, critical system data and storage locations are decentralized across multiple nodes, significantly enhancing the system’s resilience to DDoS attacks.

To prevent replay attacks, the proposed scheme employs a verification mechanism based on timestamps and one-time random numbers (nonces). For each access request, the system validates whether the timestamp falls within an acceptable time window and ensures that the nonce has not been used previously. This mechanism guarantees that, even if an attacker intercepts a legitimate access request, it cannot be reused to gain unauthorized access. Additionally, the immutability of blockchain ensures that all access records are accurately preserved, further strengthening the system’s capability to defend against replay attacks.

## 6. Experiment and Performance Analysis

This section presents an experimental analysis of the mechanism proposed in this paper. Experimental simulations were carried out in Python 3.12.2 using PyCharm 14475.56. All results from the experimental evaluations were derived using the same computer, which was equipped with an Intel(R) Core(TM) i9-13900K CPU at 5.90 GHz and 32 GB of RAM, with 31.7 GB available.

**Figure 4:**
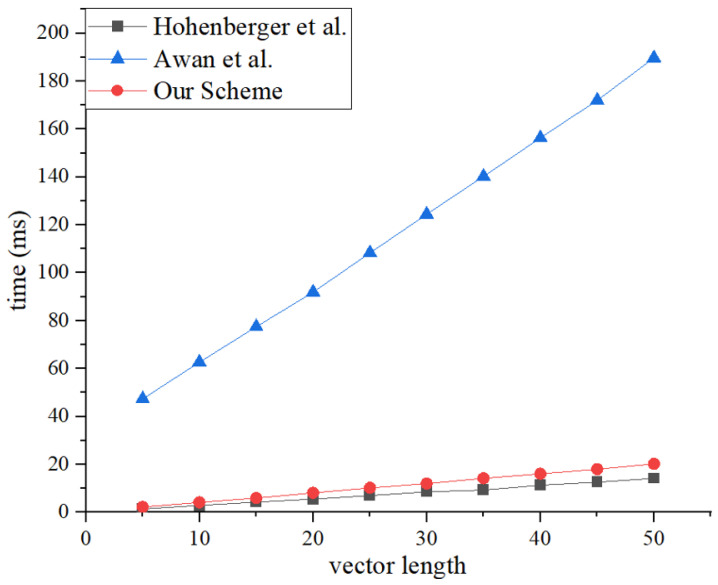
Encryption algorithm encryption time consumption.

**Figure 5:**
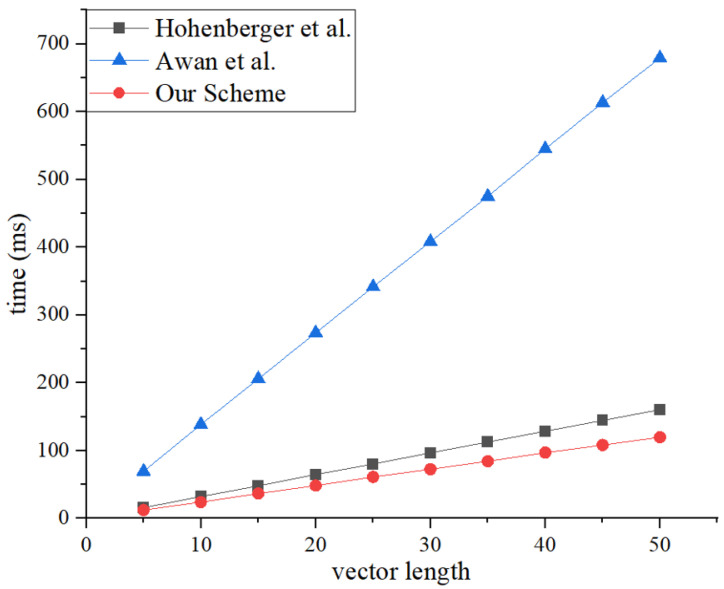
Encryption algorithm decryption time consumption.

This research averaged the results of 20 experiments to obtain the final experimental data. As shown in Figure 4 and Figure 5, the registered inner-product encryption scheme proposed in this paper has slightly higher time consumption for encryption and decryption than Hohenberger’s registered attribute encryption scheme [50]. This is because inner-product encryption inherently consumes more time than attribute encryption. However, attribute encryption carries a risk of attribute disclosure, so the additional time consumed is considered worthwhile for enhanced security. Compared to Awan’s traditional encryption scheme [31], the registered inner-product encryption scheme still has considerable efficiency advantages.

**Figure 6:**
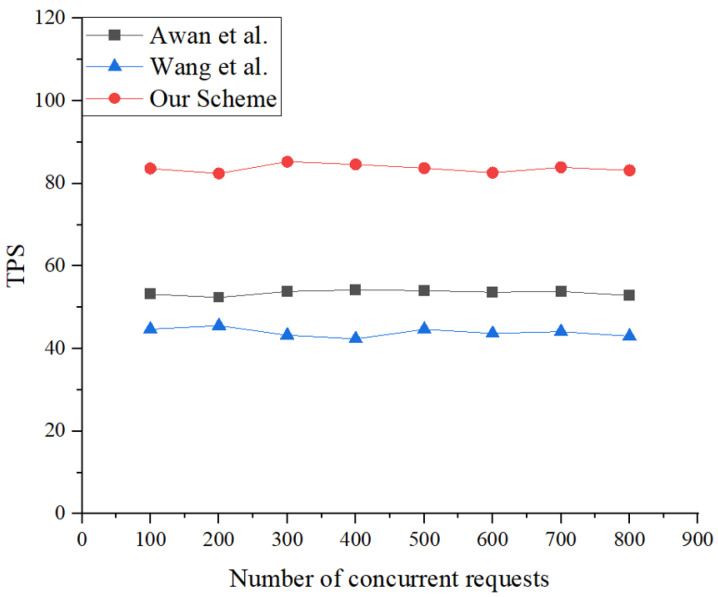
Throughput performance comparison.

Furthermore, this research assessed the system’s throughput across a spectrum of 100–800 simultaneous requests. The system’s throughput remained relatively stable due to the orderly processing of transactions facilitated by the Hyperledger Fabric platform, which is supported by a Kafka cluster. As depicted in Figure 5 and Figure 6, the throughput of the mechanism introduced in this study surpassed that of the blockchain-based zero-trust access control schemes by Awan [31] and Wang [32] under identical conditions. This improvement is due to the introduction of auxiliary chains, which significantly reduce the transaction pressure on the main chain, allowing the mechanism to better handle the more frequent and rapid data interactions of IoT devices expected in future applications.

**Figure 7:**
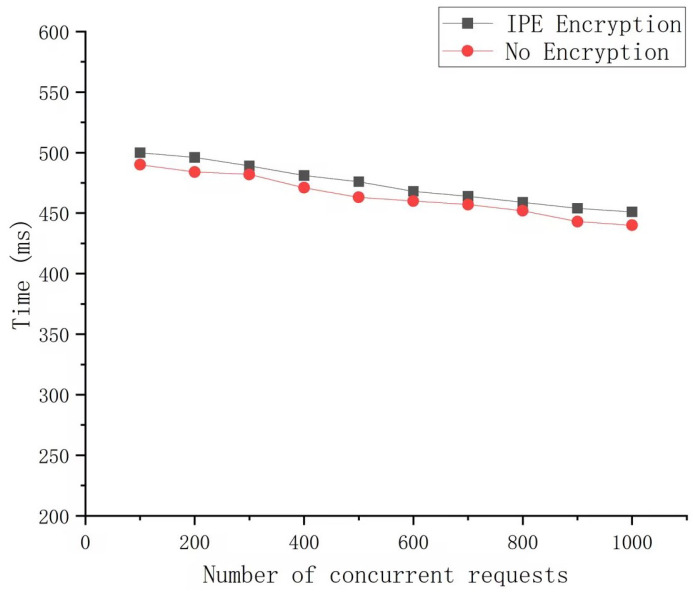
System throughput with and without encryption under varying concurrent requests.

To evaluate the impact of encryption overhead on system performance, we utilized Hyperledger Fabric (version 2.4.4) as the underlying blockchain platform and employed a Kafka cluster to enhance the sequential processing capacity of transactions. For the measurement of system throughput, Hyperledger Caliper was used as the performance testing tool. We compared the throughput of the system with and without IPE under varying levels of concurrent requests. The results, presented in Figure 7, demonstrate that the system maintains high throughput in both scenarios, with only a slight reduction in performance due to encryption.

Specifically, the throughput without encryption decreases from 500 tx/s to 451 tx/s as the number of concurrent requests increases from 100 to 1000. In comparison, the throughput with encryption decreases from 490 tx/s to 440 tx/s over the same range. This represents a performance impact of approximately 2–3%, which is minimal and demonstrates that the encryption overhead does not significantly degrade system performance.

The results also highlight the effectiveness of the framework’s distributed architecture and dynamic load balancing mechanisms, which mitigate the impact of encryption on system performance. These findings confirm that the proposed framework is capable of maintaining high efficiency and scalability while providing strong security guarantees, making it suitable for large-scale 6G IoT deployments.

In implementing self-sovereign identity management, this paper uses a secret sharing scheme to fragment and store user identity information. During the identity verification process, the identity information is reconstructed by selecting appropriate values of *n* (the total number of shares) and *k* (the minimum number of shares required to reconstruct the secret). This ensures that, without a sufficient number of shares, no one can reconstruct the complete polynomial and therefore obtain the full identity, thus ensuring the security and reliability of user identity management. To validate the system’s performance under severe security threats, this paper simulates the probability of an adversary successfully reconstructing identity information by collecting partial shares.

**Figure 8:**
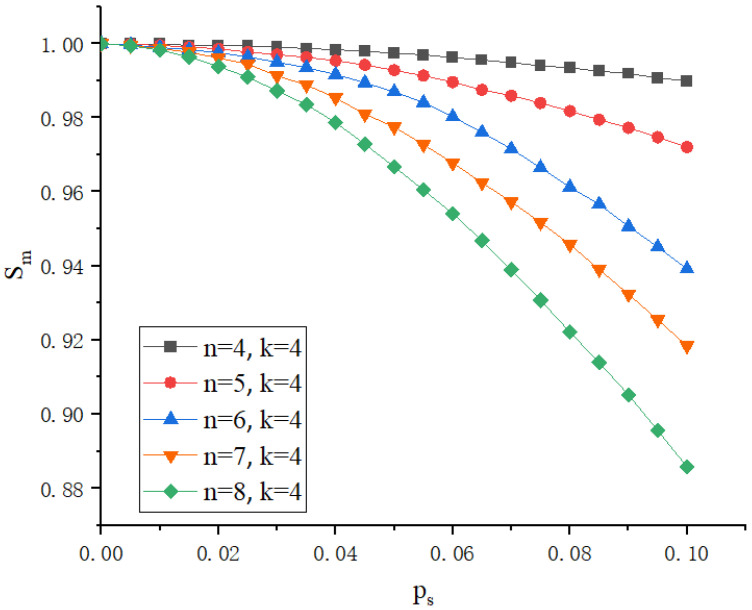
Impact of changes in the total number of identity management slices.

**Figure 9:**
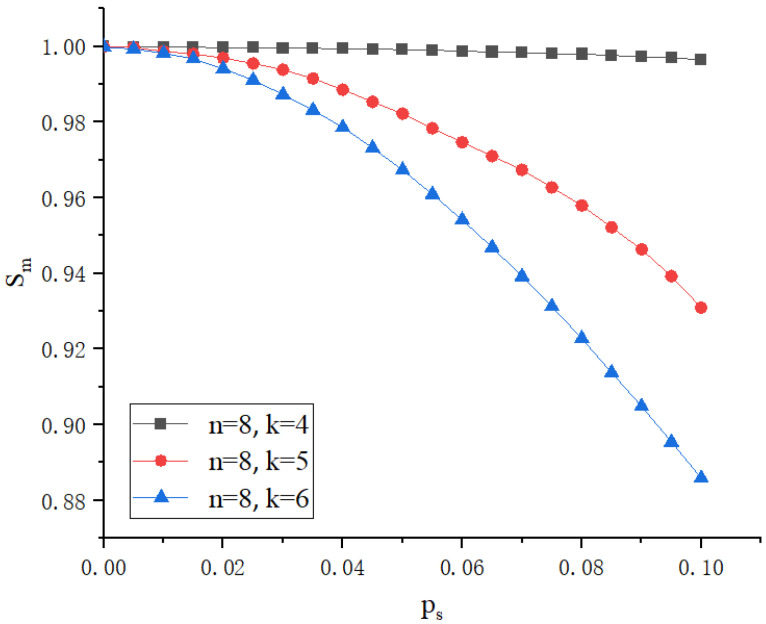
Impact of changes in the minimum number of slices for identity management.

Figure 8 and Figure 9 illustrate the security of the proposed scheme in protecting identity information under different parameter conditions, where *n* represents the total number of generated shares, *k* represents the minimum number of shares required to reconstruct the secret, $P_{s}$ represents the probability of an adversary obtaining a single share, and $S_{M}$ represents the probability of successfully defending against the adversary. Figure 8 shows the impact of different total shares *n* on system security with a fixed threshold k=4. It can be observed that, as $P_{s}$ increases, $S_{M}$ gradually decreases. Additionally, as *n* increases, even with the same $P_{s}$, the system’s security improves because the adversary needs more shares to reach the threshold *k*. Figure 9 displays how changing the value of *k* affects system security with a fixed n=8. As the *k* value increases, meaning more shares are needed to reconstruct the identity credentials, the difficulty for the adversary to succeed increases, thereby increasing the $S_{M}$ value. This indicates that higher *k* values can more effectively enhance the system’s security.

## 7. Conclusions

This paper introduces a zero-trust self-sovereign identity and access control framework that integrates blockchain technology and inner-product encryption, designed to address the security challenges in 6G-enabled IoT environments. By leveraging a registration-based inner-product encryption algorithm, fine-grained access control is achieved without the need for a centralized key generation center, significantly enhancing data security. Identity data and access logs are immutably stored on the blockchain, with sidechains used to optimize data storage efficiency and support high-speed data interactions. Micro-segmentation through trust mechanism-based confidentiality ratings further limits the scope of potential attacks within the network.

Despite these achievements, certain limitations remain. The framework’s scalability in ultra-large-scale IoT deployments and its adaptability to resource-constrained devices require further investigation. Future research will focus on enhancing the flexibility and scalability of the zero-trust model, with a particular emphasis on optimizing dynamic identity management and decentralized authentication. These enhancements aim to further align the proposed framework with the demands of emerging IoT ecosystems in 6G environments.

## Figures and Tables

**Table 1:** Functional comparison.

Features/Schemes	Traditional Centralized Schemes	Awan et al. [31]	Our Scheme
**Fine-Grained Access Control**	Coarse-grained, role-based	Role-based, partial fine-grained support	Fine-grained, attribute-based control
**Cross-Domain Support**	Weak	Strong, blockchain-dependent	Strong, with blockchain and auxiliary chains
**Data Storage and Transmission Efficiency**	Medium, centralized storage	High, limited by main chain performance	High, with auxiliary chains offloading main chain
**Flexibility of Dynamic Identity Management**	Weak	Moderate, supports partial dynamics	High, supports real-time trust value adjustment

## Data Availability

Data are contained within the article.

## References

[1] Nguyen H., Lim Y., Seo M., Jung Y., Kim M., Park W. (2023). Strengthening information security through zero trust architecture: A case study in South Korea. Proceedings of the International Conference on Intelligent Systems and Data Science.

[2] Mohseni Ejiyeh A. Real-Time Lightweight Cloud-Based Access Control for Wearable IoT Devices: A Zero Trust Protocol. Proceedings of the First International Workshop on Security and Privacy of Sensing Systems.

[3] Ren Y., Huang D., Wang W., Yu X. (2023). BSMD: A blockchain-based secure storage mechanism for big spatio-temporal data. Future Gener. Comput. Syst..

[4] Hasan K.M.B., Sajid M., Lapina M.A., Shahid M., Kotecha K. (2024). Blockchain technology meets 6 G wireless networks: A systematic survey. Alex. Eng. J..

[5] Ren Y., Leng Y., Cheng Y., Wang J. (2019). Secure data storage based on blockchain and coding in edge computing. Math. Biosci. Eng..

[6] Jahid A., Alsharif M.H., Hall T.J. (2023). The convergence of blockchain, IoT and 6G: Potential, opportunities, challenges and research roadmap. J. Netw. Comput. Appl..

[7] Nguyen D.C., Ding M., Pathirana P.N., Seneviratne A., Li J., Niyato D., Dobre O., Poor H.V. (2021). 6G Internet of Things: A comprehensive survey. IEEE Internet Things J..

[8] Wu Q., Han Z., Mohiuddin G., Ren Y. (2023). Distributed Timestamp Mechanism Based on Verifiable Delay Functions. Comput. Syst. Sci. Eng..

[9] Qadir Z., Le K.N., Saeed N., Munawar H.S. (2023). Towards 6G Internet of Things: Recent advances, use cases, and open challenges. ICT Express.

[10] Ravidas S., Lekidis A., Paci F., Zannone N. (2019). Access control in Internet-of-Things: A survey. J. Netw. Comput. Appl..

[11] Gong Y., Yao H., Liu X., Bennis M., Nallanathan A., Han Z. (2024). Computation and Privacy Protection for Satellite-Ground Digital Twin Networks. IEEE Trans. Commun..

[12] Gong Y., Yao H., Xiong Z., Chen C.P., Niyato D. (2024). Blockchain-aided digital twin offloading mechanism in space-air-ground networks. IEEE Trans. Mob. Comput..

[13] Jiang H., Ji P., Zhang T., Cao H., Liu D. (2024). Two-Factor Authentication for Keyless Entry System via Finger-Induced Vibrations. IEEE Trans. Mob. Comput..

[14] Guo F., Yu F.R., Zhang H., Li X., Ji H., Leung V.C. (2021). Enabling massive IoT toward 6G: A comprehensive survey. IEEE Internet Things J..

[15] Luo H., Zhang Q., Sun G., Yu H., Niyato D. (2024). Symbiotic blockchain consensus: Cognitive backscatter communications-enabled wireless blockchain consensus. IEEE/ACM Trans. Netw..

[16] Wang J., Chen J., Ren Y., Sharma P.K., Alfarraj O., Tolba A. (2022). Data security storage mechanism based on blockchain industrial Internet of Things. Comput. Ind. Eng..

[17] Ren Y., Leng Y., Qi J., Sharma P.K., Wang J., Almakhadmeh Z., Tolba A. (2021). Multiple cloud storage mechanism based on blockchain in smart homes. Future Gener. Comput. Syst..

[18] Sun L., Wang Y., Ren Y., Xia F. (2024). Path signature-based xai-enabled network time series classification. Sci. China Inf. Sci..

[19] Behrad S., Bertin E., Tuffin S., Crespi N. (2020). A new scalable authentication and access control mechanism for 5G-based IoT. Future Gener. Comput. Syst..

[20] Shin S., Kwon T. (2020). A privacy-preserving authentication, authorization, and key agreement scheme for wireless sensor networks in 5G-integrated Internet of Things. IEEE Access.

[21] Wang Q., Chen D., Zhang N., Qin Z., Qin Z. (2017). LACS: A lightweight label-based access control scheme in IoT-based 5G caching context. IEEE Access.

[22] Li Q., Xia B., Huang H., Zhang Y., Zhang T. (2021). TRAC: Traceable and revocable access control scheme for mHealth in 5G-enabled IIoT. IEEE Trans. Ind. Inform..

[23] Zhou Z., Gaurav A., Gupta B.B., Lytras M.D., Razzak I. (2021). A fine-grained access control and security approach for intelligent vehicular transport in 6g communication system. IEEE Trans. Intell. Transp. Syst..

[24] Wei Y., Gai K., Yu J., Zhu L., Choo K.K.R. (2024). Trustworthy Access Control for Multiaccess Edge Computing in Blockchain-Assisted 6G Systems. IEEE Trans. Ind. Inform..

[25] Saha S., Das A.K., Wazid M., Park Y., Garg S., Alrashoud M. (2024). Smart Contract-Based Access Control Scheme for Blockchain Assisted 6G-Enabled IoT-Based Big Data Driven Healthcare Cyber Physical Systems. IEEE Trans. Consum. Electron..

[26] Ma Y., Yuan Z., Li W., Li Z. (2021). Novel solutions to NOMA-based modern random access for 6G-enabled IoT. IEEE Internet Things J..

[27] Wu K., Shi J., Guo Z., Zhang Z., Cai J. (2021). Research on security strategy of power internet of things devices based on zero-trust. Proceedings of the 2021 International Conference on Computer Engineering and Application (ICCEA).

[28] Liu Y., Hao X., Ren W., Xiong R., Zhu T., Choo K.K.R., Min G. (2022). A blockchain-based decentralized, fair and authenticated information sharing scheme in zero trust internet-of-things. IEEE Trans. Comput..

[29] Fang L., Wu C., Kang Y., Ou W., Zhou D., Ye J. (2022). Zero-Trust-Based Protection Scheme for Users in Internet of Vehicles. Secur. Commun. Netw..

[30] Dhar S., Bose I. (2021). Securing IoT devices using zero trust and blockchain. J. Organ. Comput. Electron. Commer..

[31] Awan S.M., Azad M.A., Arshad J., Waheed U., Sharif T. (2023). A blockchain-inspired attribute-based zero-trust access control model for IoT. Information.

[32] Wang J., Chen J., Xiong N., Alfarraj O., Tolba A., Ren Y. (2023). S-BDS: An effective blockchain-based data storage scheme in zero-trust IoT. ACM Trans. Internet Technol..

[33] Garzon S.R., Yildiz H., Küpper A. (2022). Towards decentralized identity management in multi-stakeholder 6G networks. Proceedings of the 2022 1st International Conference on 6G Networking (6GNet).

[34] Xu J., Xue K., Tian H., Hong J., Wei D.S., Hong P. (2020). An identity management and authentication scheme based on redactable blockchain for mobile networks. IEEE Trans. Veh. Technol..

[35] Shi N., Tan L., Li W., Qi X., Yu K. (2021). A blockchain-empowered AAA scheme in the large-scale HetNet. Digit. Commun. Netw..

[36] Xiong R., Ren W., Hao X., He J., Choo K.K.R. (2023). BDIM: A Blockchain-Based Decentralized Identity Management Scheme for Large Scale Internet of Things. IEEE Internet Things J..

[37] Lu Y., Zheng X. (2020). 6G: A survey on technologies, scenarios, challenges, and the related issues. J. Ind. Inf. Integr..

[38] Ren Y., Zhu F., Wang J., Sharma P.K., Ghosh U. (2021). Novel vote scheme for decision-making feedback based on blockchain in internet of vehicles. IEEE Trans. Intell. Transp. Syst..

[39] Vaezi M., Azari A., Khosravirad S.R., Shirvanimoghaddam M., Azari M.M., Chasaki D., Popovski P. (2022). Cellular, wide-area, and non-terrestrial IoT: A survey on 5G advances and the road toward 6G. IEEE Commun. Surv. Tutor..

[40] Fang G., Sun Y., Almutiq M., Zhou W., Zhao Y., Ren Y. (2023). Distributed medical data storage mechanism based on proof of retrievability and vector commitment for metaverse services. IEEE J. Biomed. Health Inform..

[41] Ren Y., Lv Z., Xiong N.N., Wang J. (2024). HCNCT: A cross-chain interaction scheme for the blockchain-based metaverse. ACM Trans. Multimed. Comput. Commun. Appl..

[42] Ren Y., Zhu F., Sharma P.K., Wang T., Wang J., Alfarraj O., Tolba A. (2019). Data query mechanism based on hash computing power of blockchain in internet of things. Sensors.

[43] Liu C., Tan R., Wu Y., Feng Y., Jin Z., Zhang F., Liu Y., Liu Q. (2024). Dissecting zero trust: Research landscape and its implementation in IoT. Cybersecurity.

[44] He Y., Huang D., Chen L., Ni Y., Ma X. (2022). A survey on zero trust architecture: Challenges and future trends. Wirel. Commun. Mob. Comput..

[45] Syed N.F., Shah S.W., Shaghaghi A., Anwar A., Baig Z., Doss R. (2022). Zero trust architecture (zta): A comprehensive survey. IEEE Access.

[46] Yang Y., Bai F., Yu Z., Shen T., Liu Y., Gong B. (2024). An Anonymous and Supervisory Cross-Chain Privacy Protection Protocol for Zero-Trust IoT Application. ACM Trans. Sens. Netw..

[47] Buck C., Olenberger C., Schweizer A., Völter F., Eymann T. (2021). Never trust, always verify: A multivocal literature review on current knowledge and research gaps of zero-trust. Comput. Secur..

[48] Itodo C., Ozer M. (2024). Multivocal Literature Review on Zero-Trust Security Implementation. Comput. Secur..

[49] Francati D., Friolo D., Maitra M., Malavolta G., Rahimi A., Venturi D. (2023). Registered (inner-product) functional encryption. Proceedings of the International Conference on the Theory and Application of Cryptology and Information Security.

[50] Hohenberger S., Lu G., Waters B., Wu D.J. (2023). Registered attribute-based encryption. Proceedings of the Annual International Conference on the Theory and Applications of Cryptographic Techniques.

